# Leisure-Time Physical Activity and Metabolic Syndrome in Older Adults

**DOI:** 10.3390/ijerph16183358

**Published:** 2019-09-11

**Authors:** Laura Gallardo-Alfaro, Maria del Mar Bibiloni, David Mateos, Lucía Ugarriza, Josep A. Tur

**Affiliations:** 1 Research Group on Community Nutrition & Oxidative Stress, University of Balearic Islands, IDISBA &CIBEROBN (Physiopathology of Obesity and Nutrition), 07122 Palma de Mallorca, Spain; lauragala3@gmail.com (L.G.-A.); mar.bibiloni@uib.es (M.d.M.B.); david-mateos@hotmail.es (D.M.); luciaugarriza@gmail.com (L.U.); 2 Camp Redó Primary Health Care Center, IBSalut, Palma de Mallorca, 07120 Palma de Mallorca, Spain

**Keywords:** metabolic syndrome, physical activity, leisure-time, older adults

## Abstract

*Background*: Metabolic syndrome (MetS) is a cluster of risk factors for cardiovascular disease, atherosclerosis and diabetes mellitus type 2 which may be reduced by practicing regular physical activity. *Objective:* To assess the leisure-time physical activity (LTPA) of older adults with MetS and without MetS. Methods: Cross-sectional study of older adults (55–80 years old) from Balearic Islands (Spain) with MetS (*n* = 333; 55% men) and without MetS (*n* = 144; 43.8% men). LTPA was assessed with the validated Spanish version of the Minnesota LTPA Questionnaire. Two criteria of physically active were used: >150 min/week of moderate physical activity or >75 min/week of vigorous physical activity or a combination of both, and total leisure-time energy expenditure of >300 MET·min/day. Sociodemographic and lifestyle characteristics, anthropometric variables, MetS components, and adherence to the Mediterranean diet (MD) were also measured. *Results:* MetS subjects showed lower energy expenditure in LTPA, lower adherence to the MD, higher obesity and waist circumference, and were less active than non-MetS peers. LTPA increased as participants got older and there was higher LTPA intensity as educational level increased. Adherence to MD was as high as LTPA was. *Conclusions:* MetS is associated with physical inactivity and unhealthy diet. To increase LTPA recommendations and raise awareness in the population about the health benefits of PA and high adherence to MD is highly recommended.

## 1. Introduction

Metabolic syndrome (MetS) is a complex of interrelated risk factors for cardiovascular disease (CVD), atherosclerosis and diabetes mellitus type 2 (DM2) [1], responsible for two-fold increase in coronary heart disease risk, cerebrovascular disease risk, and 1.5-fold increase in all-cause mortality risk [2]. Risk factors include disglycaemia, high blood pressure, raised triglyceride levels, low high-density lipoprotein cholesterol levels, and obesity (particularly central adiposity) [2,3,4,5]. The causes responsible for the MetS seem to be multifactorial, including family history and lifestyle [6].

MetS prevalence is rising worldwide, which largely relates to an increasing inactive lifestyle, inadequate nutrition, ageing of population, and obesity [1,3]. As obesity prevalence doubled in the last three decades, MetS prevalence increased in parallel [7]. The average prevalence of MetS is nearly 35% of all adults and 46.7% of those aged 60 years or older [8]. MetS is now both a public health and clinical problem.

Physical inactivity has been recognized as a global pandemic, representing one of the most pressing public health problems of the 21st century [9]. According to the World Health Organization (WHO), physical inactivity has been identified as the fourth leading risk factor for global mortality [10,11]. The evidence shows that practicing regular physical activity (PA), with at least 150 min of moderate-intensity aerobic PA per week or 75 min of vigorous-intensity aerobic PA per week, or an equivalent combination of moderate-and vigorous-intensity PA [10], improves insulin sensitivity, reduces hypertriglyceridemia, improves fibrinolytic ability, and decreases blood pressure as well as the risk of developing major cardiovascular and metabolic diseases [12,13]. In addition, exercise intensity is an important factor reversing the risk factors of the MetS [14]. Regular, moderate and vigorous PA has been demonstrated to prevent MetS [14,15,16]. Light PA enhances energy expenditure, which is associated with a lower prevalence of MetS [6]. Even a recent increase in PA level can have an important effect on health [17]. In this way, PA is related to healthy aging [18,19,20] and reduces the likelihood of developing MetS [21]. Consequently, an inactive lifestyle, in which people spend a large amount of their day sitting or lying down [22] is associated with increased mortality compared to a physically active lifestyle [23].

On the other hand, The Mediterranean Diet (MD) is characterized by a high consumption of fruits and nuts, vegetables, legumes and cereals, a high intake of olive oil as the principal source of dietary lipids, a low intake of saturated lipids and meat, a moderate intake of fish, a low-to-moderate intake of dairy products and a wine consumption in low to moderate amounts [24]. Furthermore, there is strong evidence that the adherence to a Mediterranean dietary pattern (MDP) is associated with a healthier status, due to the protective effect of MD against several chronic diseases, including CVD, several cancers and total mortality [24,25,26,27]. Moreover, adherence to MD has shown to be inversely associated with MetS [28,29].

In this way, practicing regular PA and high MD adherence seem to be important contributors to reduce the incidence of MetS. Therefore, the main objective of this study is to assess the leisure-time physical activity (LTPA) of older adults according to the presence or absence of MetS.

## 2. Methodology

### 2.1. Study Design, Sample and Ethics

This is a cross-sectional study to assess the effect of lifestyle factors on the health of older adults living in Balearic Islands, Spain. The study population was 477 participants, men (52% of participants) aged 55–80 years and women aged 60–80 years with no previously documented cardiovascular disease engaged in social and municipal clubs, health centers, and sport clubs. Exclusion criteria included being institutionalized, suffering from a physical or mental illness which would have limited their participation in physical fitness assessment or their ability to respond to questionnaires, chronic alcoholism or drug addiction and intake of drugs for clinical research over the past year.

The study protocol and procedures were performed according to the ethical standards of the Declaration of Helsinki, and were approved by the Ethics Committee of Research of Balearic Islands (CEIC-IB2251/14PI and CEIC-IB1295/09PI). All participants provided written informed consent prior to participation.

### 2.2. Socioeconomic and Lifestyle Determinants

Sociodemographic and lifestyle characteristics were collected from each participant. Educational level was ranked into primary school studies, secondary school studies and university graduate. Civil status was ranked into single, married, divorced and widow/er. Finally, information related to individual medical history, current medication use and smoking status were also obtained.

### 2.3. Anthropometric Measurements

Anthropometric variables were measured by trained personnel to minimize the inter-observer coefficients of variation. Height was measured using a wall-mounted stadiometer, to the nearest millimeter, with the subject’s head in the Frankfurt plane. Weight was measured with high-quality electronic calibrated scales. Participants were weighed in bare feet and light clothes, subtracting 0.6 kg for their clothes. Body mass index (BMI) was calculated as weight in kilograms divided by the square of height in meters (kg/m^2^). Obesity was defined as BMI ≥ 30 kg/m^2^ [2,30]. Waist circumference (WC) was measured halfway between the last rib and the iliac crest by using an anthropometric tape. Blood pressure was measured with a validated semi-automatic oscillometer (Omron HEM-705CP) after 5 min of rest in-between measurements while the participant was in a seated position. All anthropometric variables were determined in duplicate, except for blood pressure (in triplicate).

### 2.4. Metabolic Syndrome Classification

Samples of fasting blood were collected from antecubital vein after an overnight fast. Biochemical analyses were performed on fasting plasma glucose, total cholesterol, low high-density lipoprotein cholesterol (HDL-c) and triglyceride (TG) concentrations in local laboratories using standard enzymatic methods. Participants were classified as “with MetS (MetS)” (*n* = 333) and “without MetS (non-MetS)” (*n* = 144) according to the updated harmonized definition of the International Diabetes Federation and the American Heart Association and National Heart, Lung and Blood Institute [3].

### 2.5. Mediterranean Diet Adherence

The Mediterranean Dietary Pattern (MDP) was assessed according to a previously defined score indicating the degree of adherence to the traditional MD [25,31]. This Mediterranean dietary score (MDS) was converted to relative percentage of adherence using a previously described method [32,33] that is briefly summarized. An energy-adjusted value was obtained for each individual for the daily consumption of legumes, cereals (including bread and potatoes), fruit, vegetables, meat (including meat products), and milk (including dairy products). Information about the consumption of all these food items was obtained from the 137-item food-frequency questionnaire (FFQ), repeatedly validated in Spain [34]. For each item, a typical portion size was included and consumption frequencies were registered in 9 categories that ranged from “never or almost never” to “≥6 times/day”. Energy and nutrient intakes were calculated as frequency multiplied by nutrient composition of specified portion size for each food item, using a self-made computerized program based on available information in Spanish food composition tables [35]. Monounsaturated fatty acids (MUFA):Saturated fatty acids (SFA) ratio was calculated. In order to score “moderate alcohol consumption”, a transformation centred at the level of consuming 30 g/day for men (30—(30-absolute alcohol intake)), and 20 g/day for women (20—(20-absolute alcohol intake)) was used to obtain the highest value for men consuming 30 g/day or women consuming 20 g/day and progressive lower values as the consumption was lower or higher than these values. All these values were standardized as a Z value [33,36]. Total MDS was computed by adding up all the *Z *scores obtained for the favourable or more Mediterranean dietary components (legumes, cereals and roots, fruit, vegetables, fish, moderate alcohol, MUFA:SFA ratio) and subtracting the *Z *value obtained from the consumption of meat and milk:

∑ Z = Z_legumes_ + Z_cereals and roots_ + Z_fruit_ + Z_vegetables_ + Z_fish_ + Z_moderate alcohol_ + Z_MUFA:SFA_ − Z_meat_ − Z_milk_ (1)

The MDP was converted to relative percentage of adherence using the range of values of the sample. A participant with a maximum value of adherence in the sample obtained 100% of adherence. A participant with a minimum value of adherence obtained 0% in the relative percentage [32,33].

(2) $$\mathrm{Adherence}\;\left({\mathrm{Percentage}}_{i}\right)\;=\;\frac{\left(\sum^{\;}{\;Z}_{i}\;-\;\sum^{\;}\;\mathrm{Zmin}\right)*100}{\left(\sum^{\;}\;\mathrm{Zmax}\;-\;\sum^{\;}\;\mathrm{Zmin}\right)}$$

Once the percentage of adherence to the MDP was calculated, the variables that could determine a higher or lower adherence were assessed. Low Mediterranean Diet (MD) adherence was defined as a percentage of adherence below the 25th percentile, medium adherence was defined as a percentage of adherence between the 25th and 75th percentile and high adherence was defined as a percentage of adherence above the 75th percentile. 

### 2.6. Physical Activity

PA was assessed using the validated Spanish version of the Minnesota Leisure Time Physical Activity Questionnaire [37,38], a valid instrument with good validity and reliability to measure the quantity and quality of PA performed in the last year [37,38,39]. Moreover, this questionnaire is considered a useful tool for estimating the amount of PA performed in elderly people [40,41]. This questionnaire, administered by interview with trained research assistants, measures leisure time physical activities (LTPA) including household activities, performed in the last year. This questionnaire was used to assess PA quality (light-moderate and vigorous) by using metabolic equivalents of task (MET) [42]. Light-moderate activity was defined as a MET intensity of PA lower and equal to 6 METs and vigorous activity was defined as a MET intensity of PA above 6 METs [43,44]. METs were calculated by multiplying the duration spent on that activity (measured in minutes) and their respective MET intensity levels. The MET score can be obtained from tables (the Compendium of Physical Activities) [44] that show the intensity of each activity relative to resting. MET·hour/week spent on PA refers to the energy expenditure that is spent on activities, over and above existing levels of resting energy expenditure.

### 2.7. Criteria to Determine Active Population

Two criteria were used to determine “physically active” population. First criterion is based on the “Global recommendations on PA for health” by the WHO. Therefore, active population was defined as practicing at least 150 min of moderate-intensity aerobic physical activity throughout the week, or doing at least 75 min of vigorous-intensity aerobic PA throughout the week, or an equivalent combination of moderate- and vigorous-intensity activity [10]. Lower PA was understood as inactive lifestyle. A second criterion established active population as expending above 300 MET·min/day, which is related with lower heart attack risk [45,46].

### 2.8. Statistical Analyses

Analyses were performed with the Statistical Package for the Social Sciences version 25.0 (IBM SPSS Statistics for Windows, Chicago, IL, USA). Categorical variables were presented as frequencies and/or proportions. Significant differences in prevalence were calculated by means of chi-squared test or Fisher’s exact test. Continuous variables were presented as mean and standard deviation (SD) or median and interquartile range (IQR). Normality of data was assessed using Kolmogorov–Smirnov test. For normally distributed data, comparisons between two comparison groups were tested by unpaired Students’ *t*-test. Equality of variances was assessed with Levene’s test. For non-normally distributed data, the Mann-Whitney U test was used to compare the median of two independent groups. Comparisons above 2 groups were tested by the Kruskal-Wallis test and/or the Mann Whitney U test, applying the Bonferroni correction. Correlations between LTPA and MD adherence were calculated using the Spearman rank correlation. Results were considered statistically significant if *p*-value (2 tailed) <0.05.

## 3. Results

Table 1 shows general characteristics of MetS and non-MetS participants. MetS group showed higher weight, BMI and waist circumference, as well as higher percentage of men, obese participants and smokers. MetS group also showed lower adherence to the MD, compared with the non-MetS group.

Comparison of the energy expenditure in LTPA between MetS and non-MetS participants is shown in Table 2. There were differences between MetS and non-MetS subjects in total energy expenditure in LTPA in all variables, except for single and obese participants. In non-MetS participants there were differences in total activity in BMI, which was also observed in vigorous activity besides civil status, age and gender. In light-moderate activity there were differences in gender, age and educational level. In MetS group there were differences in total activity in age, educational level and MD adherence, which were also observed in light-moderate PA. In vigorous PA there were differences in gender, education level MD adherence and BMI. Furthermore, in MetS group, total LTPA was correlated with MD adherence (*r* = 0.214, *p* < 0.001), but not in non-MetS group (*r* = 0.008, *p* = 0.925). 

Percentage of active and inactive population in MetS and non-MetS according to two criteria is shown in Table 3. MetS participants were less active than non-MetS. Considering the first criterion for active population (>150 min/week of moderate PA or >75 min/week of vigorous PA), there were differences between MetS and non-MetS in gender, married, 55–64 year-old, primary and secondary education, medium MD adherence and non-obesity (<30 kg/m^2^) participants. In addition, there were higher percentages of active participants as age increased. According to the second criterion (>300 MET min/day), there were differences between MetS and non-MetS in all variables except for single and obese (≥30 kg/m^2^) participants. Furthermore, in MetS group there were differences in percentage of active people according to age and adherence to MD. 

## 4. Discussion

The main finding of this study was the low energy expenditure in LTPA of MetS participants on most studied outcomes. LTPA increased as participants got older and there was higher LTPA intensity as educational level increased. MetS subjects also showed lower adherence to the MDP than non-MetS peers; and adherence to MD was as high as LTPA was. 

Practicing regular PA is indicative of greater longevity and reduced risk of coronary heart disease, CVD, stroke and colon cancer [47]. LTPA reduces all causes and CVD death risk [48], as well as MetS development. According to the second criterion, it is important to note that adherence to MD increased in the MetS group as much as the energy expenditure in LTPA and the percentage of active participants did. This result was consistent with a previous study in which as total LTPA increased, the adherence to MD did too [49]. Furthermore, it was found that older adults with unhealthy diet tended to engage in more inactive lifestyle than older adults with a healthy diet [50].

Age is also an outcome to be considered. MetS participants who were 65–69 years old showed higher light-moderate PA levels than those aged 55–64 years. A plausible explanation could be that older people were retired, and they had more time to spend outside, increasing LTPA. This finding is contrary to a previous study pointing out that older participants were less active [51]. MetS subjects in our study showed higher levels of light-moderate PA, but lower vigorous PA than a previous study in Spain [52].

Despite that total and light-moderate PA of MetS subjects showed no differences between sexes, vigorous PA of male MetS subjects showed higher LTPA. No differences were found between sexes in percentage of physically active participants for both criteria, which is contrary to previous findings in which females were less active than men for LTPA [51].

Regarding educational level, MetS participants with university studies showed higher vigorous PA levels, which is related to lower mortality risk [53]. However, higher light-moderate PA levels were observed in MetS participants with primary and secondary education. As educational level increased, LTPA intensity did. Vigorous LTPA has been associated with lower risk of developing MetS [54]. Moreover, it was concluded that lower education level increased the incidence of mortality from cardiovascular disease [55]. Another previous study concluded that lower education was related with decreased LTPA among older adults [56]. On the contrary, our study showed no differences in percentage of active people in both groups regarding educational level, suggesting that as educational level increased, the LTPA intensity did, but not the percentage of active people. 

Obesity is associated with numerous comorbidities such as CVD, type 2 diabetes, hypertension and several cancers [57,58]. Our results showed that MetS and non-MetS obese subjects showed no differences in energy expenditure in LTPA and in percentage of active population. These results were consistent with previous findings pointing out that higher inactive leisure time and lower PA were associated with greater prevalence of increased BMI [59], as well as that insufficient LTPA was related with higher BMI [60].

MetS subjects showed low adherence to the MD compared with non-MetS participants. It is important to highlight that MD is characterized by high consumption of vegetables, legumes, fruits and nuts and whole cereals, high intake of olive oil, but low-to-moderate consumption of dairy products, low intake of meat and poultry and regular, but moderate intake of wine [31,36,61]. Accordingly, MD is recommended to improve glycaemia and cardiovascular risk factors [62], with protective effect on cardiovascular diseases [63], also being a possible therapy for MetS [64]. It was previously concluded that as adherence to MD decreases, MetS increases, resulting in a worse profile of plasma inflammation markers [36]. Moreover, lower MD adherence was observed among patients with MetS, as well as an inverse association between adherence to MD and prevalence of MetS components [65].

In our study, physically active population, MetS subjects were more inactive than non-MetS peers after the two WHO criteria were considered [10]. According to the first criterion for active population (>150 min/week of moderate PA or >75 min/week of vigorous PA or a combination of both), the MetS group showed 89.2% active participants, in contrast with 100% in the non-MetS group. Our results agree a previous study using the same recommendations, but although LTPA increased, overweight or obesity increased and physical fitness decreased [66]. Considering the second criterion (>300 MET·min/day) [46], there were 62.5% MetS active participants and 93.1% non-MetS active participants; however, our study reported higher percentage of active people among MetS participants than previous related studies [46,67].

## 5. Strengths and Limitations

The main strength of this study was that, to our knowledge, it is the first study comparing LTPA in older adults with and without MetS. However, the study has several limitations. First, this cross-sectional study limits the ability to elucidate a causal relationship between MetS and lower LTPA. Second, given that with the LTPA it is not possible to measure occupational activity, comparison of total physical activity among age groups is also limited. Finally, it is a quasi-experimental design.

## 6. Conclusions

MetS subjects showed lower energy expenditure in LTPA, were less active, with lower adherence to the MD and higher obesity and waist circumference than non-MetS peers. MetS is then associated to physical inactivity and unhealthy diet. To increase LTPA recommendations and raise awareness in the population about the health benefits of PA and high adherence to MD is highly recommended.

## Figures and Tables

**Table 1:** Baseline characteristics of study sample between MetS and non-MetS participants.

General Characteristics	MetS (*n* = 333)	Non-MetS (*n* = 144)	*p-*Value
Men, *n* (%)	183 (55.0)	63 (43.8)	**0.028**
Age, y	64.9 ± 5.4	65.5 ± 5.6	0.342
Weight, kg	86.2 ± 14.0	69.1 ± 12.5	**<0.001**
Height, cm	163.1 ± 9.3	162.4 ± 8.8	0.482
BMI, kg/m^2^	32.4 ± 3.87	26.1 ± 3.3	**<0.001**
Waist circumference, cm	108.8 ± 11.4	85.4 ± 10.9	**<0.001**
Obesity, *n* (%)	238 (94.8)	13 (5.2)	**<0.001**
Civil status, *n* (%)			0.137
Single	16 (5.0)	5 (3.5)	
Married	251 (77.7)	101 (70.1)	
Divorced	22 (6.8)	13 (9.0)	
Widow/er	34 (10.5)	25 (17.4)	
Education level, *n* (%)			0.179
Primary	153 (47.4)	68 (47.2)	
Secondary	104 (32.2)	50 (34.7)	
University	66 (20.4)	26 (18.1)	
Mediterranean Diet Adherence, *n* (%)			**<0.001**
Low (<p 25)	97 (29.1)	22 (15.3)	
Medium (p 25–p 75)	167 (50.2)	72 (50.0)	
High (>p 75)	69 (20.7)	50 (37.7)	
Smoker, *n* (%)	46 (13.9)	9 (6.3)	**0.017**

Abbreviations: BMI: body mass index; MetS: Metabolic Syndrome; p: percentile. Difference in means between MetS and without MetS group were tested by unpaired Students’ *t* test for normally distributed variables and by the Mann-Whitney U test for non-normally distributed data. Differences in percentages were tested by chi-squared test. Significant differences have been highlighted in bold.

**Table 2:** Energy expenditure (MET hour/week) in leisure-time physical activity (LTPA) between MetS (*n* = 333) vs. non-MetS (*n* = 144) participants by sex, age, civil status, education level, adherence to the Mediterranean diet (MD) and body mass index (BMI). Physical activity is divided in light-moderate activity (<6 MET) and vigorous activity (≥6 MET).

	Total Activity					Light-Moderate Activity					Vigorous Activity				
	MetS		Non-MetS		*p-*Value	MetS		Non-MetS		*p-*Value	MetS		Non-MetS		*p-*Value
	Mean ± SD	Median (IQR)	Mean ± SD	Median (IQR)		Mean ± SD	Median (IQR)	Mean ± SD	Median (IQR)		Mean ± SD	Median (IQR)	Mean ± SD	Median (IQR)	
Total	60.5 ± 48.0	45.9 (63.0)	103.1 ± 140.1	83.9 (46.2)	**<0.001**	56.7 ± 47.2	43.9 (60.3)	84.6 ± 136.4	68.6 (42.3)	**<0.001**	3.8 ± 9.2	0.9 (4.3)	18.5 ± 27.9	9.0 (17.6)	**<0.001**
Gender															
Men	60.8 ± 49.9	45.6 (60.8)	122.8 ± 207.5	83.5 (57.1)	**<0.001**	55.7 ± 49.0	42.3 (59.8)	93.4 ± 203.9 ^l^	61.4 (54.3)	**0.005**	5.1 ± 11.7 ^l^	1.9 (4.7)	29.4 ± 37.7 ^l^	10.1 (34.8)	**<0.001**
Women	60.2 ± 45.7	46.0 (62.9)	87.7 ± 34.0	84.3 (44.3)	**<0.001**	57.8 ± 45.1	45.6 (60.6)	77.6 ± 30.6 ^l^	73.4 (38.4)	**<0.001**	2.3 ± 4.1 ^l^	0.9 (3.1)	10.1 ± 11.2 ^l^	4.7 (13.1)	**<0.001**
Age															
55–64	49.6 ± 41.8 ^a,b^	36.9 (59.3)	120.1 ± 204.9	86.2 (61.7)	**<0.001**	45.7 ± 40.3 ^a,b^	34.4 (58.7)	92.0 ± 201.6 ^b^	60.0 (43.2)	**<0.001**	3.9 ± 10.7	1.4 (4.6)	28.1 ± 36.8 ^b^	10.2 (33.6)	**<0.001**
65–69	73.6 ± 50.7 ^a^	59.6 (73.3)	88.5 ± 43.3	81.2 (43.1)	**0.037**	69.2 ± 49.7 ^a^	52.7 (68.6)	75.5 ± 35.9	75.6 (37.3)	0.157	4.4 ± 8.6	1.4 (4.7)	13.0 ± 17.0	5.4 (16.7)	**<0.001**
≥70	66.2 ± 52.1 ^b^	51.3 (50.8)	90.8 ± 35.9	82.8 (42.5)	**<0.001**	63.4 ± 52.6 ^b^	48.0 (46.8)	82.8 ± 33.5 ^b^	76.5 (38.5)	**<0.001**	2.8 ± 5.7	0.9 (2.9)	8.0 ± 7.8 ^b^	5.0 (12.5)	**<0.001**
Civil status															
Single	54.5 ± 33.5	43.69 (50.3)	384.4 ± 723.0	71.9 (848.6)	0.445	53.0 ± 33.2	43.7 (48.4)	371.0 ± 715.8	62.9 (824.8)	0.612	1.6 ± 2.0	0.5 (4.3)	13.4 ± 15.5	5.2 (29.0)	0.066
Married	64.1 ± 50.5	51.2 (62.1)	96.7 ± 50.5	88.4 (48.1)	**<0.001**	59.8 ± 49.7	45.0 (60.6)	76.1 ± 39.0	72.8 (45.2)	**<0.001**	4.3 ± 10.2	1.4 (4.7)	20.6 ± 28.5 ^c^	9.6 (22.4)	**<0.001**
Divorced	39.5 ± 31.9	26.1 (64.8)	93.8 ± 38.7	87.9 (56.1)	**<0.001**	36.4 ± 31.0	22.7 (63.0)	65.7 ± 30.2	59.2 (53.1)	**0.009**	3.1 ± 4.8	2.1 (4.7)	28.1 ± 41.6	15.8 (33.3)	**0.005**
Widow/er	50.5 ± 38.9	41.2 (70.1)	77.6 ± 32.2	67.4 (46.2)	**0.001**	48.6 ± 39.1	36.0 (71.8)	71.2 ± 30.9	65.1 (32.4)	**0.003**	1.9 ± 3.1	0.7 (2.9)	6.3 ± 10.0 ^c^	3.7 (3.6)	**0.002**
Education level															
Primary	68.5 ± 51.4 ^d^	57.0 (69.9)	100.4 ± 54.5	94.2 (46.6)	**<0.001**	65.8 ± 50.9 ^d^	54.6 (71.3)	81.7 ± 37.4 ^e^	78.0 (45.7)	**0.001**	2.7 ± 5.3 ^d^	0.9 (2.8)	18.7 ± 33.5	7.6 (13.2)	**<0.001**
Secondary	58.2 ± 45.8	44.7 (60.1)	115.1 ± 228.3	73.4 (48.2)	**<0.001**	53.9 ± 44.3	40.6 (56.1)	96.9 ± 226.9 ^e^	58.5 (44.7)	**0.01**	4.3 ± 13.1 ^f^	1.2 (4.6)	18.2 ± 24.4	6.6 (25.8)	**<0.001**
University	42.7 ± 30.5 ^d^	36.5 (49.4)	87.0 ± 39.8	85.7 (49.6)	**<0.001**	36.7 ± 26.8 ^d^	31.1 (34.7)	68.3 ± 34.8	62.1 (40.4)	**<0.001**	6.0 ± 9.7 ^d f^	3.3 (6.6)	18.8 ± 16.7	13.2 (29.4)	**<0.001**
Mediterranean Diet Adherence															
Low (<p 25)	42.3 ± 37.9 ^i,k^	33.8 (40.4)	170.3 ± 340.9	82.1 (90.7)	**<0.001**	38.5 ± 36.5 ^i,k^	28.7 (40.4)	453.2 ± 337.3	66.1 (71.1)	**<0.001**	3.9 ± 13.3 ^i,k^	0.5 (3.4)	17.1 ± 24.1	7.0 (19.8)	**<0.001**
Medium (p 50–75)	65.8 ± 48.2 *^i,j^*	53.6 (67.2)	89.9 ± 47.0	86.1 (42.0)	**<0.001**	62.1 ± 47.8 ^i,j^	50.7 (64.5)	71.8 ± 37.6	69.2 (39.5)	**0.023**	3.7 ± 7.2 ^i,j^	0.9 (4.4)	18.1 ± 26.9	9.4 (19.6)	**<0.001**
High (p > 75)	72.0 ± 53.5 ^k,j^	58.6 (76.2)	92.5 ± 43.5	82.8 (52.9)	**0.004**	67.8 ± 52.2 ^k,j^	54.6 (72.1)	72.7 ± 32.1	70.6 (54.4)	0.137	4.1 ± 6.5 ^k,j^	2.3 (4.7)	19.8 ± 31.2	9.4 (17.1)	**<0.001**
BMI															
<30 kg/m2	59.2 ± 38.9	48.3 (50.5)	106.4 ± 145.9 ^g^	88.0 (46.4)	**<0.001**	53.7 ± 37.0	43.9 (54.3)	87.0 ± 142.5	68.7 (43.6)	<0.001	5.5 ± 8.7 ^h^	2.8 (5.7)	19.4 ± 28.6 ^g^	9.4 (17.5)	**<0.001**
≥30 kg/m2	61.1 ± 51.6	45.5 (68.6)	69.8 ± 45.3 ^g^	65.3 (34.5)	0.253	58.0 ± 51.1	43.8 (67.1)	60.0 ± 35.3	61.7 (43.3)	0.407	3.1 ± 9.3 ^h^	0.9 (2.8)	9.8 ± 17.4 ^g^	1.1 (10.6)	0.178

Abbreviations: BMI: body mass index; IQR: Interquartil range; MD: Mediterranean Diet; MET: Metabolic equivalent; MetS: Metabolic Syndrome; p: percentile; SD: Standard deviation. Difference in means for intensity of physical activity (total physical activity, light-moderate activity, heavy activity) between MetS and non-MetSwere tested by the Mann Whitney U test. Differences among categories of the same group were tested by the Kruskal-Wallis test and/or the Mann Whitney U test, applying the Bonferroni correction. ^a^ Differences (*p-value* < 0.05) between 55–64 years vs. 65–69 years old in the same group (MetS). ^b^ Differences *(p-value* < 0.05) between 55–64 years vs. ≥70 years old in the same group (non-MetS or MetS). ^c^ Differences *(p-value* < 0.05) between married vs. widow/er in the same group (non-MetS). ^d^ Differences *(p-value* < 0.05) between primary studies vs. university studies in the same group (MetS). ^e^ Differences *(p-value* < 0.05) between primary studies vs. secondary studies in the same group (non-MetS). ^f^ Differences *(p-value* < 0.05) between primary studies vs. university studies in the same group (MetS). ^g^ Differences *(p-value* < 0.05) between <30 kg/m^2^ vs. ≥30 kg/m^2^ in the same group (non-MetS). ^h^ Differences *(p-value* < 0.001) between <30 kg/m^2^ vs. ≥30 kg/m^2^ in the same group (MetS). ^i^ Differences *(p-value* < 0.05) between low MD adherence vs. medium MD adherence in the same group (MetS). ^j^ Differences *(p-value* < 0.05) between high MD adherence vs. medium MD adherence in the same group (MetS). ^k^ Differences *(p-value* < 0.05) between low MD adherence vs. high MD adherence in the same group (MetS). ^l^ Differences between gender in the same group. Significant differences have been highlighted in bold.

**Table 3:** Percentage of active and inactive population in MetS (*n* = 288) and non-MetS (*n* = 142) participants according to two criteria (1st Criterion: >150 min/week of moderate physical activity or >75 min/week of vigorous physical activity; 2nd Criterion: >300 MET min/day).

	1st Criterion					2nd Criterion				
	MetS (*n* = 333)		Non-MetS (*n* = 144)		*p-*Value	MetS (*n* = 333)		Non-MetS (*n* = 144)		*p-*Value
	Active	Inactive	Active	Inactive		Active	Inactive	Active	Inactive	
Total	257 (89.2)	31 (10.8)	142 (100)	0 (0)	**<0.001**	180 (62.5)	108 (37.5)	134 (93.1)	10 (6.9)	**<0.001**
Gender										
Men	139 (88.5)	18 (11.5)	62 (100)	0 (0)	**0.002**	96 (61.1)	61 (38.9)	57 (90.5)	6 (9.5)	**<0.001**
Women	118 (90.1)	13 (9.9)	80 (100)	0 (0)	**0.002**	84 (64.1)	47 (35.9)	77 (95.1)	4 (4.9)	**<0.001**
*p-*value	0.675		1.000			0.603		0.283		
Age										
55–64	117 (84.2)	22 (15.8)	62 (100)	0 (0)	**<0.001**	71 (51.4)	67 (48.6)	59 (92.2)	5 (7.8)	**<0.001**
65–69	82 (93.2)	6 (6.8)	46 (100)	0 (0)	0.094	65 (73.9)	23 (26.1)	42 (91.3)	4 (8.7)	**0.022**
≥70	58 (95.1)	3 (4.9)	34 (100)	0 (0)	0.550	44 (71.0)	18 (29.0)	33 (97.1)	1 (2.9)	**0.002**
*p-*value	0.026		1.000			**0.001**		0.567		
Civil status										
Single	14 (93.3)	1 (6.7)	5 (100)	0 (0)	1.000	10 (66.7)	5 (33.3)	4 (80.0)	1 (20.0)	1.000
Married	200 (89.7)	23 (10.3)	99 (100)	0 (0)	**<0.001**	145 (65.0)	78 (35.0)	93 (92.1)	8 (7.9)	**<0.001**
Divorced	16 (80.0)	4 (20.0)	13 (100)	0 (0)	0.136	7 (35.0)	13 (65.0)	13 (100)	0 (0)	**<0.001**
Widow/er	27 (90.0)	3 (10.0)	25 (100)	0 (0)	0.242	18 (60.0)	12 (40.0)	24 (96.0)	1 (4.0)	**0.003**
*p-*value	0.551		1.000			0.064		0.428		
Education level										
Primary	120 (90.2)	13 (9.8)	66 (100)	0 (0)	**0.005**	90 (67.7)	43 (32.3)	62 (91.2)	6 (8.8)	**<0.001**
Secondary	79 (91.9)	7 (8.1)	50 (100)	0 (0)	**0.047**	53 (61.6)	33 (38.4)	48 (96.0)	2 (4.0)	**<0.001**
University	50 (84.7)	9 (15.3)	26 (100)	0 (0)	0.052	30 (50.8)	29 (49.2)	24 (92.3)	2 (7.7)	**<0.001**
*p-*value	0.365		1.000			0.085		0.587		
Mediterranean Diet Adherence										
Low (p < 25)	68 (84.0)	13 (16.0)	22 (100)	0 (0)	0.065	38 (47.5)	42 (52.5)	21 (95.5)	1 (4.5)	**<0.001**
Medium (p 25**–**p 75)	133 (90.5)	14 (9.5)	70 (100)	0 (0)	**0.006**	99 (66.9)	49 (33.1)	66 (91.7)	6 (8.3)	**<0.001**
High (p > 75)	56 (93.3)	4 (6.7)	50 (100)	0 (0)	0.084	43 (71.7)	17 (28.3)	47 (94.0)	3 (6.0)	**0.003**
*p-*value	0.162		1.000			**0.004**		0.787		
BMI										
<30 kg/m^2^	82 (92.1)	7 (7.9)	129 (100)	0 (0)	**0.002**	61 (69.3)	27 (30.7)	123 (93.9)	8 (6.1)	**<0.001**
≥30 kg/m^2^	175 (87.9)	24 (12.1)	13 (100)	0 (0)	0.370	119 (59.5)	81 (40.5)	11 (84.6)	2 (15.4)	0.084
*p-*value	0.288		1.000			0.113		0.209		

Abbreviations: BMI: body mass index; MD: Mediterranean Diet; MetS: Metabolic Syndrome; p: percentile. Data is shown as *n* (%). Differences in percentages between MetS and non-MetS participants and in different variables of the same group were tested by chi-squared test or Fisher’s exact test. Significant differences have been highlighted in bold.
